# Initial Recommendations for Virtual Reality in Mental Health Care from the American Medical Extended Reality Association

**DOI:** 10.1177/29941520251394885

**Published:** 2025-11-18

**Authors:** Jessica Stone, Leslie Baker, Rachel A. Altvater, Triton Ong

**Affiliations:** ^1^ Jessica Stone Ph.D. LLC, Fruita, Colorado, USA.; ^2^ Therapy2Thrive®, Ruby Hill Marriage and Family Counseling Center, Inc., Pleasanton, California, USA.; ^3^ Creative Psychological Health Services, Catonsville, Maryland, USA.; ^4^ Doxy.me Inc., Charleston, South Carolina, USA.

**Keywords:** virtual reality, mental health care, clinical recommendations, ethics, immersive technology

## Abstract

Mental health care has long been a leading domain for the application of clinical virtual reality (VR) technologies. The recent proliferation of affordable all-in-one VR devices has significantly increased accessibility and created new opportunities for innovative mental health therapies. As immersive technologies become more integrated into daily life, scientific evidence and clinical insights can be synthesized into actionable recommendations. The American Medical Extended Reality Association, in collaboration with the Mental Health Virtual Reality International Coalition, presents these comprehensive recommendations for the use of VR in clinical mental health care. This work summarizes the current state of the field, examines the supporting scientific literature, outlines ethical considerations and their practical applications, and offers research-based and experience-informed recommendations. Additionally, it identifies key collaborative directions to advance the responsible and effective use of VR in mental health care. These recommendations may serve as a foundational resource for clinicians, researchers, developers, and policymakers for the integration of VR in mental health practice.

## Introduction

Nearly 1 billion people were living with a mental health disorder in 2019.^1^ Mental health disorders account for more than $5 trillion USD in economic impacts, lost productivity, and health care costs with more than 418 million years of lost human life annually.^2^ Depression, anxiety, post-traumatic stress disorder (PTSD), psychological distress, and sleep problems increased more than 20% during the COVID-19 pandemic.^3^ Mental health burdens were particularly harsh for health care workers, nearly half of whom reported symptoms of PTSD, anxiety, and psychological distress.^4^ For decades, health care workers have faced unsustainable workloads and escalating burnout with no substantive interventions in sight.^5^ Mental health providers are experiencing unprecedented burnout as demand for therapy continues to reach record levels while thousands of clinicians have been leaving the field.^6^ Governments and health care systems around the world have changed policies to simplify reimbursement and promote the scalability of mental health care with technological solutions.^7^

Virtual reality (VR) is one such technology that can enhance provider logistics and client experiences in mental health care. In the context of health care, VR is a system that completely envelopes the user’s visual and sometimes auditory field to focus the user’s perception in a computer simulation.^8^ The combination of these simulation, display, and sensor technologies creates experiences unique to VR: immersion, perceiving the simulation as “real”^9^; presence, feeling as if you are “there” in the simulated place^10^; copresence, feeling that simulated others are “here” with you^11^; embodiment, feeling that a simulated body is “you”^12^; and telepresence, feeling that simulated experiences with simulated others are really happening.^13^ VR-enhanced therapies have been well-demonstrated to improve client engagement, provider efficiency, and outcomes in a growing variety for mental health conditions for both clinical and general populations.^14–17^ Dedicated and enterprise VR systems are available for clinical mental health care, in addition to an increasing variety of nonclinical VR applications that may be useful in therapy.^18–21^ VR technologies are also growing in popularity, affordability, and maturity. Meta, Apple, HTC, Google, Samsung, Sony, and other large consumer technology companies are competing to establish VR devices, software, and marketplaces.^22–24^ Advances in consumer technology and growing clinical evidence have positioned mental health care as a leading application of VR technologies. However, only 0.1–13% of mental health therapists have adopted VR in their practices.^25–27^

It is clear that VR can enhance evidence-based mental health care,^14,16,28^ but mental health therapists report feeling untrained on using VR in practice and unable to find information that is accessible or reputable.^29–31^ Therapists need guidance in the form of advice from experts and peer therapists,^29^ endorsement by professional organizations,^32^ and accessible evidence and tutorials.^33^ Toward these ends, we formed the Mental Health Virtual Reality International Coalition (MHVR-IC) to share insights between professionals in mental health care, research, education, and product development. In collaboration with the American Medical Extended Reality Association (AMXRA) and the Journal of Medical XR, the MHVR-IC assembled these preliminary recommendations in preparation for the development of formal guidelines on the application of VR in mental health care.

The authors—members of both AMXRA and MHVR-IC—assembled these recommendations in a collaborative process. Coauthors Stone, Baker, and Altvater have each been practicing clinical mental health care for 15–34 years and using VR with clients for 5–15 years, while coauthor Ong has been publishing peer-reviewed research on VR health care for 9 years. We first discussed personal experiences with VR, VR protocols used in clinical and research practice, and organized insights into themes. We then expanded each theme with recommendations from recent research literature. We present preliminary recommendations for the use of VR in mental health care by reviewing guiding principles for mental health providers, applying these principles to VR in mental health care, and then discussing paths to formalize these recommendations into clinical guidelines and best practices.

### Guiding principles for VR in mental health care

In the mental health fields, each professional organization maintains codes of conduct to guide clinical practices. Mental health providers should follow existing guidance from their respective practice associations. For example, in the United States, the American Psychological Association (APA),^34^ American Counseling Association,^35^ National Association of Social Workers,^36^ and American Association for Marriage and Family Therapy^37^ maintain codes of conduct and licensure standards for mental health professionals. Here, we will use the APA General Principles as an aspirational guide for VR in mental health care since it is recognized, well-established, and widely applicable.^34^

### Beneficence and nonmaleficence: First, do no harm

Mental health professionals work to safeguard those they support. While VR is a promising therapeutic tool, many aspects of its long-term impact and effectiveness remain unknown. With VR, the two most common risks are related to perceptual discomfort (e.g., dizziness, nausea, or disorientation following the use of a VR headset) or physical injury (e.g., falling or bumping into objects while vision is occluded by a headset).^38^ Both risks can be mitigated if not prevented with proper preparation.^39^ There are concerns that children may be susceptible to VR risks such as eye development, head or neck pain, cognitive distortions, and addiction.^40^ While such concerns have not been evidenced in the clinical research literature, we recommend mental health professionals proceed with caution, consult with peers and the most up-to-date clinical evidence, and evaluate continuously.

### Fidelity and responsibility: Seek training, supervision, and ethical practice

Mental health care providers aim to practice within their scope of expertise, maintain professional conduct, and represent their capabilities accurately. The transformative potential of VR brings risks if applied improperly. Unethical or untrained use can result in harm, such as retraumatization or a breakdown of trust in the therapeutic process. Proper training and consultation empower clinicians to practice safely, minimize risks, and maintain transparency. The immersive nature of VR demands of practitioners unique technical competence, ethical responsibility, and a commitment to ongoing education.

### Integrity: Be trustworthy

Accuracy, honesty, and truthfulness are conspicuously important in mental health care and often make the difference in therapeutic relationships with clients. For the use of VR in treatment, it is important to be transparent about competence and knowledge gaps. VR has been extensively demonstrated for exposure therapy for anxiety conditions^41–43^ and specific forms of pain management.^44,45^ There is promising preliminary evidence supporting VR for mindfulness and relaxation.^46–48^ For most other mental health needs, clinical evidence for VR therapy is in exploratory phases.^15^ VR is a novel therapeutic intervention, so clarity about the level of understanding and the proposed outcome is recommended.

### Justice: Be fair and help clients overcome unfairness

People seeking mental health services deserve quality care regardless of personal characteristics or economic conditions. The people who would benefit most from technology are often the last to access it. Sliding scales are common in mental health care and may be considered with VR services as well. Older models of VR headsets may also meet the clinical needs at fairer cost with minimal impact on logistics. Therapists and clients may rent headsets at lower, as-needed costs compared with the purchase of a personal headset. Many modern VR experiences follow design conventions of entertainment video games. As a result, even clinical VR apps can have a steep learning curve for those without sophisticated video gaming experience. It is best to thoroughly and repeatedly introduce clients to VR systems with no assumptions of prior experience.

### Respect for rights and dignity: Meet clients where they are

Mental health care professionals use proper safeguards to protect the vulnerable and seek perspective into each client’s unique experience. It is critical to ensure implementation of VR for mental health care is safe and secure, whether the services are provided on-site, virtually, or asynchronously. Practitioners who use VR are underrepresented in the research literature. To enhance knowledge of VR in supporting a wide range of individuals, mental health professionals are encouraged to participate in research opportunities and share their experiences through various publications such as case studies, conferences, and social media.

### Moving ahead with intent

A VR experience that is beneficial for one person could be detrimental for another. In one study, the family-friendly VR short film “Pearl” was heartwarming and nostalgic to most participants, anxiety-inducing for some who anticipated a sudden crash, and caused undue emotional distress for one participant who lost a loved one in a car collision.^49^ As VR becomes increasingly common in mental health care, foundational principles can help clinicians to navigate the evolving landscape of VR interventions. Ongoing education, transparency about the current limits of VR research, and a commitment to individualized care will help ensure that VR is applied in ways that enhance client well-being (Table 1). Careful implementation and continuous assessment of VR in clinical settings will allow for innovative and ethical therapeutic practices that prioritize client health and integrity.

**Table 1.** General Recommendations for Virtual Reality in Clinical Mental Health Care

1. *Obtain informed consent.* Provide clients with clear explanations of the VR procedures, equipment, benefits, and potential risks. For clients under the age of consent, obtain consent from the client’s legal guardian in addition to the client’s assent. Provide information about the hardware and software to be used, confidentiality considerations, considerations for the client’s needs, and how VR aligns with the therapeutic goal. 2. *Tailor VR content for individual client needs.* Rule out VR contraindications in advance (e.g., prone to severe vertigo). Select VR hardware and software that are developmentally appropriate. Avoid content that could cause undue anxiety or trauma responses unless specifically intended for therapeutic exposure. Monitor and adjust treatment plans continuously to assess the impact of VR on the client’s well-being. 3. *Develop technical proficiency continuously.* Practitioners can become familiar with VR systems, hardware, software, and accessories before use with clients. This ensures they can guide clients and provide technical support if issues arise. Gain knowledge and hands-on experience with VR systems and understand their clinical applications. 4. *Seek legal counsel*. As appropriate, acquire input from experienced legal professionals to review relevant terms of service. Become fluent enough to arrange proper contracts and discuss concerns with clients confidently. 5. *Engage in supervision and peer case review.* Supervision allows clinicians to reflect on challenging cases, refine use of VR, and address uncertainties with peers and experts. Building networks and engaging with professional organizations fosters the exchange of experiences and best practices, contributing to the growth of knowledge for VR in mental health care.

VR, virtual reality.

### Incorporating VR into mental health care

**FIG. 1.**
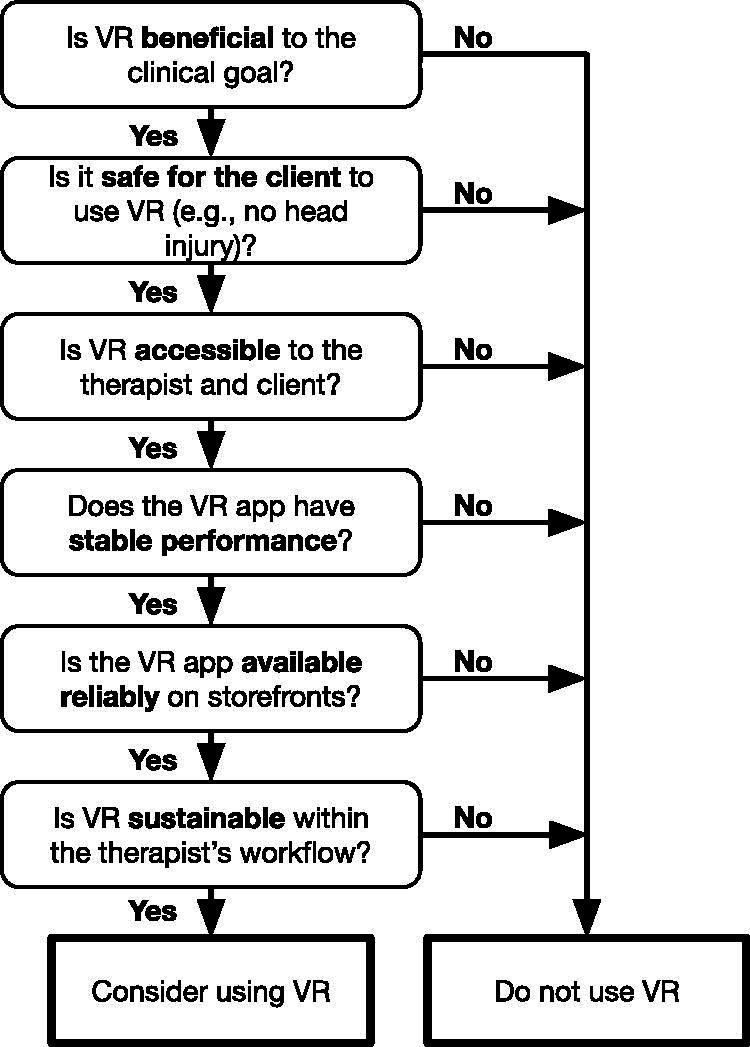
Considerations for using VR in mental health care. VR, virtual reality.

When considering VR in mental health care, it is essential to use a flexible, client-centered approach to account for individual needs and preferences (Fig. 1). We provide the following recommendations derived from the authors’ collective experiences and recent research on implementation of VR and its side effects.^29,39,50,51^

### Start with the client’s unique needs

#### Needs assessment

Consider the client’s presenting concerns, therapeutic goals, and emotional readiness for immersive interventions. For example, a client struggling with social anxiety might benefit from VR simulating public speaking or networking events. Evaluate their readiness to face these challenges, starting with less intimidating scenarios before progressing.

#### Functional capacity

VR experiences may be tailored to the client’s cognitive and emotional maturity, avoiding approaches, which may be overly complex or abstract for the individual. Account for physical or sensory limitations that might impact the client’s ability to engage with VR. For example, clients with limited mobility may prefer seated or stationary VR experiences that do not require turning, walking, or precise controller inputs.

### Match the intervention to the client

#### Therapeutic context

VR can complement traditional therapy and does not replace existing evidence-based and first-line interventions. For example, a client with trauma may benefit from VR as an adjunct to psychoeducation or grounding techniques.

#### Skill-building

VR can simulate real-world scenarios for clients to acquire and practice generalizable skills such as emotional regulation, communication, or problem-solving. For example, a client learning anger management might practice calming responses during a VR environment replicating conflict at work or home.

#### Exposure therapy

VR can provide safe, gradual exposure to controllable stimuli if the content and pacing are aligned with the client’s emotional tolerance. For example, a client with PTSD may use VR to revisit specific trauma cues incrementally, under the therapist’s guidance after establishing trust and stability.

#### Relaxation and stress management

Immersive mindfulness or relaxation environments can help clients reduce stress or practice grounding techniques with instant, constant, and visible feedback. For example, a client experiencing chronic stress might engage in guided VR meditation in a virtual forest, incorporating biofeedback for real-time monitoring of their breathing and heart rate.

### Select VR equipment to meet the goal

**FIG. 2.**
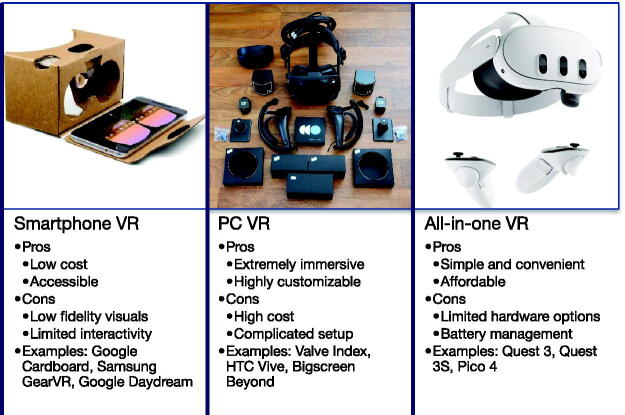
Benefits, downsides, and examples of modern VR headset options.

Modern VR headsets generally fall into one of three categories: smartphone-based, personal computer (PC)-based, and all-in-one. Each category comes with specific benefits and trade-offs with features to facilitate care (Fig. 2).

*Smartphone VR* uses the client’s smartphone as a display when inserted into a simple VR frame. Smartphone VR is widely accessible with headsets purchasable for as little as $5 when combined with a smartphone the client already owns. Smartphone VR may be preferable when affordability is a priority and high-fidelity VR is not essential to the clinical goal. Given its limited immersion and reduced sensory experience, we advise using smartphone VR in settings where deep interactivity is not required.

*PC VR* can provide highly detailed visuals and the most immersive full-body tracking available, contributing to a deep sense of engagement in therapeutic settings. However, PC VR setups require expensive equipment, often including wall-mounted camera systems and many cables to manage. These systems are ideal for clients who already own high-end VR equipment or for clinics with space and budget to accommodate high-end VR therapy needs.

*All-in-one VR*, such as Meta Quest devices, offers the portability of smartphone VR with some of the immersive qualities of PC VR, at a reasonable price point. Haptic feedback in all-in-one headsets further enhances the sense of engagement, enabling clinicians to leverage tactile responses during therapeutic exercises. However, at the time of this writing (mid 2025), only HTC, Pico, and Meta are manufacturing all-in-one VR headsets. Both Pico and Meta have faced scrutiny for data privacy concerns,^52^ and Pico headsets may face potential restrictions in the United States.^53^ As a result, we currently advise using Meta headsets (e.g., Quest 2, Quest 3, or Quest 3S) with clear understanding of their privacy policies, as well as the privacy policies of each respective VR app.

While there are creative ways to incorporate off-the-shelf VR apps and games into treatment, many therapists may prefer dedicated clinical solutions. XRHealth, Virtually Better, PsyTechVR, RealizedCare, OxfordVR, and other platforms offer comprehensive VR therapy services for headset management, training, technical support, and app personalization. Some such platforms have been evaluated clinically and negotiated coverage with public and private health insurance, addressing important barriers, so therapists can focus on immersive care.

### Navigate insurance reimbursement

**FIG. 3.**
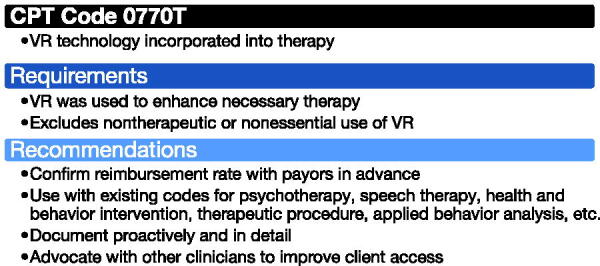
Recommendations for reimbursement strategies for VR mental health services.

Reimbursement for VR services is evolving, particularly as VR gains broader adoption in mental health care. Navigating these complexities requires clinicians to understand current policies, leverage emerging coding systems, and advocate for VR’s value in therapeutic settings (Fig. 3).

#### Stay updated on local and national policies

In the United States, emerging recommendations and policies offer opportunities to integrate VR into reimbursable services. Resources such as the Nixon Law Group’s guide to VR reimbursement and the American Medical Association’s insights into Current Procedural Terminology (CPT) coding updates provide valuable frameworks for navigating reimbursement systems.^54,55^

#### Understand and utilize CPT code 0770T

The 0770T code was designated for reporting VR services used in a therapeutic context. This allows providers to bill for VR sessions as standalone services or as part of broader therapeutic interventions.^56^ Providers may document the use of VR in alignment with payor requirements, clearly linking the technology to therapeutic goals and outcomes to maximize rates.

#### Advocate for reimbursement

When payors are hesitant, therapists can demonstrate VR’s clinical efficacy by presenting evidence of its ability to enhance client engagement, improve outcomes, and reduce long-term costs. For instance, VR’s capacity to simulate real-life scenarios in a controlled and immersive environment can accelerate therapeutic progress for clients with social anxiety or phobias.

#### Document proactively

Use detailed session notes to show how VR aligns with therapeutic goals. Include specific metrics, such as changes in anxiety levels or skill acquisition milestones, to strengthen claims. Track usage of the 0770T code and payor responses to build institutional knowledge and refine reimbursement strategies.

### Do due diligence

Using VR in clinical practice requires careful evaluation and preparation to ensure alignment with therapeutic goals and a safe, effective experience for clients. Clinicians may explore the VR software and hardware in advance, evaluating each for clinical utility and sensitivities, which might be activated by the experiences. Rehearsing first-time user experiences, navigating menu systems, and troubleshooting common technical issues are essential steps to guide clients and ensure a smooth introduction to VR with confidence. Be aware of flashing lights, periods of darkness, sudden loud sounds, and potentially frightening characters or scenarios. A careful review process allows therapists to make informed decisions about appropriateness for individual clients, ensuring interventions are both effective and considerate of therapeutic needs. Clinicians can stay informed through research on emerging VR technologies, attend trainings to refine their skills, and seek supervision or consultation when integrating new tools into practice. Collaborative discussions with peers or supervisors can provide valuable insights into program selection and client-specific considerations, ensuring ethical and effective use of VR.

### Introduce clients to VR therapy

A client’s first experience with VR can heavily influence their expectations for therapeutic outcomes. For this reason, we believe clients’ introduction to VR should be done gradually with ample pretraining to ensure confidence and proficiency with headsets, controllers, safety, apps, interfaces, and experiences (Table 2).^57^

**Table 2.** Recommendations for Introducing Clients to Virtual Reality for Mental Health Care

**Introduce the client to the VR equipment** 1. Orient the client to the VR headset, controllers, cables, and sensors (if applicable). 2. Explain the headset before placement, noting it will obstruct view of their surroundings. 3. Explain the hand tracking, controller buttons, triggers, and joysticks. 4. Discuss ways to exit a program from menus and by simply lifting the headset. 5. Ensure the client understands the arrangement of VR within the room. Emphasize clearance in all directions, including overhead. **Prepare the client to enter VR** 6. Consider seated VR experiences until the client is confident enough to stand or move around. 7. Arrange tactile indicators to help the client maintain awareness of their position in the room. For example, a secure rug underfoot or fan blowing from a fixed direction. 8. Help the client understand and use VR safety features (e.g., boundaries or passthrough). 9. Consider installing glasses spacers if the client cannot switch to other vision correction such as contact lenses. **Teach the client how to use the controllers** 10. Place the controllers in the client’s hands. Secured the safety straps around each wrist. 11. Identify each button, trigger, and joystick to the client. Consider intuitive instructions rather than abstract button labels (e.g., “pointer finger trigger” instead of “L2,” “use your right thumb to press the top button” instead of “B”). **Place the VR headset on the client (“donning”)** 12. With permission, place the headset on the client’s head. Avoid snagging hair, piercings, and head accessories. 13. Allow the client to hold the headset in place while the therapist adjusts straps for fit. 14. Use verbal explanations during adjustments (e.g., “I am going to tighten the dial on the back of the headset”) and ask the client to indicate their comfort during fitment. 15. Consider starting the VR app before placing the headset on the client to minimize their need for menu navigation. **Initiate VR therapy and engage the client** 16. Monitor VR use by watching the client’s perspective using features such as casting, a linked headset, or join within a multiplayer environment. 17. Ensure the therapist is an active participant in the process, avoiding a one-sided or isolative experience where the client may feel alone in the VR environment/experience.

### Manage risks in VR

**FIG. 4.**
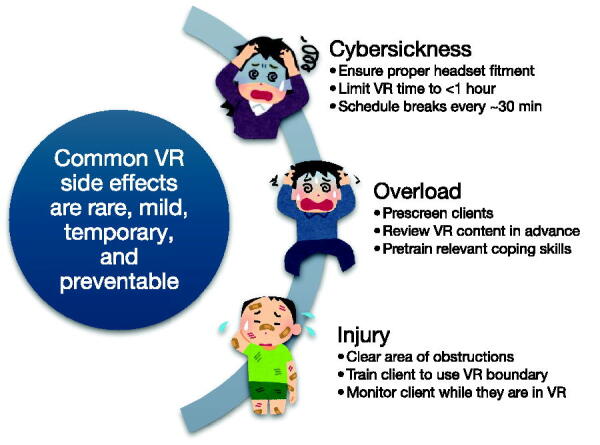
Descriptions and mitigation strategies for common VR side effects.

VR experiences can be emotionally evocative. Like any clinical tool, its use requires thoughtful planning to minimize potential risks and ensure positive outcomes. While adverse effects such as depersonalization, dissociation, or overstimulation are rare when sessions are structured, clinicians should remain mindful of these possibilities and adopt proactive mitigation strategies (Fig. 4).

### Known short-term risks of VR

#### Cybersickness

Some clients may experience disorienting symptoms such as dizziness or nausea resulting from VR use, particularly with prolonged sessions or poorly adjusted equipment.^58^ Ensure the VR headset is at the proper head and face placement, the lenses are spaced correctly, and the fit is comfortably snug. Incorrect headset fit can cause painful pinching and tugging, excessive headset movement, and visual instability—all of which can increase the likeliness of cybersickness.

#### Psychological overload

Immersive environments may evoke strong emotional responses, which can feel overwhelming during emotionally evocative experiences such as exposure therapy.^59^ These risks can be minimized by thoroughly vetting VR software experiences and prescreening clients for content and sensitivities.

#### Physical injury

Control the environment to ensure the therapy space is free from hazards during VR use. Hazards may include obstacles to movement, furniture with sharp edges, and objects that may snag the client or VR equipment such as light fixtures overhead or unsecured rugs underfoot.

### Unknown long-term risks of VR

#### Depersonalization and dissociation

For clients predisposed to dissociation or those processing complex trauma, immersive virtual environments may temporarily heighten feelings of detachment or altered perception of self.^60^ These effects can be mitigated by carefully selecting content, managing session duration, and providing grounding exercises before and after use.

#### Emotional desensitization

Clients exposed to intense virtual scenarios repeatedly may experience reduced sensitivity to similar real-world experiences.^61^ Proper pacing and integration into a broader therapeutic framework can help generalize in-headset experiences to a client’s day-to-day interactions.

#### Behavioral and cognitive fatigue

Extended or overly frequent VR sessions can lead to fatigue, affecting emotional regulation and concentration.^62^ Managing session duration and incorporating breaks can help prevent overstimulation.

### Consider assigning self-guided VR

Older adolescent and adult clients may be good candidates for self-guided use of VR tools between sessions (e.g., homework), depending on the nature of the therapy and individual needs. However, unsupervised use of VR during therapy can result in clients experiencing counter-therapeutic content. We advise instructing clients to only use designated VR programs during programmatic sessions with direct clinical supervision unless the client stands to benefit from unsupervised use of VR, is generally fluent with VR operations, and is not at high risk for distress or self-harm.

### Support clients as they exit VR

**FIG. 5.**
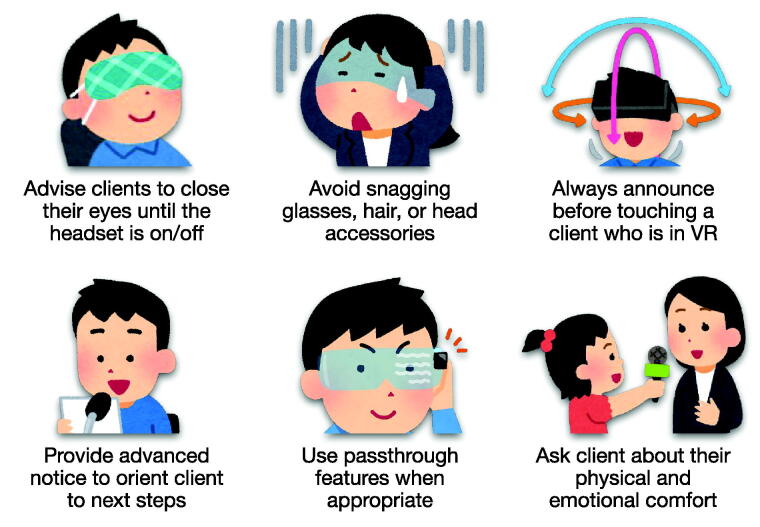
Recommendations for helping clients don and doff VR equipment.

The process of ending a VR experience, removing the headset, and transitioning back to physical reality (“doffing”) is a critical part of the therapeutic experience. This transition could possibly affect their emotional state, physical orientation, and ability to process what they experienced. Clinicians are advised to provide structured support to ensure the client feels grounded, safe, and able to integrate the VR session into their broader therapeutic goals (Fig. 5).

#### Transition from VR to reality gradually

Avoid ending VR sessions abruptly. Allow clients time to prepare for the exit by signaling when the session is nearing its conclusion. For example, “we have five more minutes in this environment. Take a moment to finish the task and start bringing your attention back to the physical room.”

#### Ground and reorient the client

Once the headset is removed, help clients reorient to their physical surroundings. This may involve a brief pause to check for any lingering physical effects, such as dizziness or disorientation. Encourage the client to look around the room, feel their feet on the ground, and take slow, deep breaths to anchor themselves in the present.

#### Check their physical and emotional condition

Ask clients how they are feeling physically and emotionally immediately after the session. Address any discomfort, such as cybersickness or emotional distress, with appropriate interventions.

### Provide extra support for clients using VR over telehealth

For telehealth, additional precautions may be important as the therapist may not have direct access to the client before, during, or after the session. Ensure the client has a reliable means of communicating throughout the session (e.g., keeping a smartphone video call connected despite meeting in VR). Encourage clients to set up a safe and private space for their VR session, free from distractions, to facilitate smoother transitions. Have a plan in place with responsive options local to the client. Consider inviting the client to involve others local to themselves such as family or roommates, if desired.

### Sanitize VR equipment after each use

**FIG. 6.**
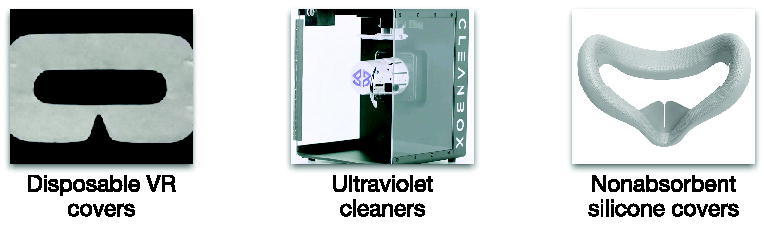
Accessories to promote hygiene and sanitation of VR equipment.

Sanitation protocols are vital whether VR equipment is owned personally by the client or shared within the clinic. Although rare, VR headsets have been found to harbor unpleasant or harmful microorganisms such as *Staphylococcus*, *Moraxella*, and *Micrococcus*.^63,64^ More commonly, VR experiences can evoke intense reactions, making it likely that headsets will accumulate sweat or tears. Single-use, disposable VR covers are available for respective VR headsets. Ultraviolet cleaners such as Cleanbox have been utilized in health care settings.^65^ These passive means of sanitation are convenient for situations in which VR headsets are passed between individuals rapidly. However, deep manual cleanings are still necessary for proper sanitation, even for personal VR headsets used by a single individual. We recommend following the cleaning instructions of the headset manufacturer, as some common sanitizing procedures may damage critical components of VR lenses, sensors, or surfaces. We also recommend silicone covers and head straps, as well as sweat bands for more vigorous use cases. While slightly less comfortable than foam or cloth surfaces, silicone coverings allow for easy and fast-drying wipedown using common sanitizing wipes. These aftermarket accessories can improve the comfort and usability of VR headsets, provide a waterproof barrier between absorbent surfaces and the user’s body, and promote sanitation (Fig. 6).

### Closing the gaps in knowledge, practice, and research

Several critical needs must be addressed to make VR easier to adopt, more sustainable to use in clinical practice, and more impactful for client outcomes.

We understand little about how mental health providers and their clients are using VR. As a result, lack of actionable and reputable information remains a leading barrier for mental health providers interested in adopting or even learning about VR therapy.^31,66,67^ It is essential that clinicians participate in research and share insights through accessible channels such as open access research publications, professional conferences, educational courses, and social media. Therapists who use VR in their mental health services have been difficult to find and engage in such efforts historically. In 2020, researchers conducted a thorough search of VR therapists in the United States and found contact information for only 128 therapists, of whom only 17 participated in the study.^68^ Dissemination of VR is likely to remain challenging until the experiences of frontline VR providers are visible and accessible to researchers, clients, payors, and fellow clinicians. Similarly, commercial solutions will struggle to mature until the market potential is clear for VR in mental health care. Of particular value would be the collaborative creation of standardized VR consent forms and tutorials for clinical implementation.^21,69,70^ Toward these ends, outreach and dissemination efforts are currently underway within professional organizations such as the AMXRA (amxra.org), International Virtual Reality Healthcare Association (ivrha.org), and MHVR-IC (discord.gg/ZkCRAXta). Open collaboration, transparent participation, and curation of publicly accessible information will be vital to the advancement of VR.

The ways people are using VR for mental health are rapidly outpacing clinical practice and empirical research. People have been using the VRChat social video game as a form of self-guided therapy to combat loneliness and depression, explore their identities, and reduce social and phobic anxieties.^71–74^ Others have reported playing immersive exergames such as Beat Saber, Supernatural, and Thrill of the Fight to relieve psychological distress.^75–77^ While clinically unsupervised self-treatment is potentially risky,^40,78^ the mass appeal of these VR games can inspire innovative care from creative therapists. The success of these consumer entertainment games provides valuable proof of concept for innovative mental health treatments with VR. Exposure therapy for social anxiety, for example, is notoriously difficult to arrange in-person due to the logistics and privacy concerns of conducting therapy in uncontrolled public spaces.^79–81^ Social VR games such as VRChat let people interact in public or private spaces, with layers of privacy through pseudonymous usernames and VR avatar disguises. This type of VR game includes features that may signal the potential to enhance mental health care in ways only possible in VR. Customizable VR avatars may permit therapists to inhabit the likeness of another to enhance therapy for grief and trauma processing.^82–84^ The interaction between VR environments and the user’s inputs are also potential paths to new forms of mental health care, such as the nonpharmacological elicitation of psychedelic experiences.^85–87^ The unique properties of VR can empower mental health care with unparalleled personalization, repeatability, comfort, and control. It will be important to approach these potentials with rigor and caution.

Immersive technologies are undergoing an arms race as the world’s largest consumer technology companies compete for market share. The fundamental distinctions between virtual, augmented, and mixed reality continue to blur as emerging headsets combine features in ways not previously possible. These are exciting times for VR and signal pressing needs for ongoing surveillance of immersive technologies, use cases, and ethical issues. VR seems positioned to intersect with emerging metaverse platforms, which may provide access to pervasive and interconnected benefits as well as risks to social and physical development.^88–90^ It is likely that people will spend more time in VR, more often, for more business and personal purposes.^91,92^ Toward these near futures, we present these recommendations as preliminary with the intent to add, remove, and update its contents progressively.

### Planned updates to these recommendations

These recommendations provide a broad approach to the use of VR in mental health care, informed by both the authors’ clinical experiences and recent research selections. As VR in mental health care continues to evolve, these recommendations will be updated in collaboration with AMXRA, practicing mental health providers, and other professional organizations to reflect emerging research, regulatory developments, and technological advancements. We intend to continue formal exploration of VR in mental health care using mixed methods approaches, such as combining qualitative interviews with quantitative surveys of VR therapy experts as well as non-VR therapists. Refinements to these recommendations resulting from such research would then enable Delphi studies to establish expert consensus guidelines such as operational definitions, design requirements of VR hardware, curated lists of clinical and nonclinical VR software, and best practices. We look forward to collaboration in preparing for a future in which mental health care and VR become increasingly interlinked.

## Abbreviations Used

AMXRA: American Medical Extended Reality Association
APA: American Psychological Association
CPT: Current Procedural Terminology
MHVR-IC: Mental Health Virtual Reality International Coalition
PC: personal computer
PTSD: post-traumatic stress disorder
VR: virtual reality
